# Reversible control of the magnetization of spinel ferrites based electrodes by lithium-ion migration

**DOI:** 10.1038/s41598-017-12948-6

**Published:** 2017-10-02

**Authors:** Guodong Wei, Lin Wei, Dong Wang, Yanxue Chen, Yufeng Tian, Shishen Yan, Liangmo Mei, Jun Jiao

**Affiliations:** 1 School of Physics and State Key Laboratory of Crystal Materials, Shandong University, Jinan, 250100 P. R. China; 2 School of Microelectronics, Shandong University, Jinan, 250100 P. R. China; 3 Department of Mechanical and Materials Engineering, Portland State University, Post Office Box 751, Portland, Oregon, 97207-0751 United States

## Abstract

Lithium-ion (Li-ion) batteries based on spinel transition-metal oxide electrodes have exhibited excellent electrochemical performance. The reversible intercalation/deintercalation of Li-ions in spinel materials enables not only energy storage but also nondestructive control of the electrodes’ physical properties. This feature will benefit the fabrication of novel Li-ion controlled electronic devices. In this work, reversible control of ferromagnetism was realized by the guided motion of Li-ions in MnFe_2_O_4_ and γ-Fe_2_O_3_ utilizing miniature lithium-battery devices. The *in-situ* characterization of magnetization during the Li-ion intercalation/deintercalation process was conducted, and a reversible variation of saturation magnetization over 10% was observed in both these materials. The experimental conditions and material parameters for the control of the ferromagnetism are investigated, and the mechanism related to the magnetic ions’ migration and the exchange coupling evolution during this process was proposed. The different valence states of tetrahedral metal ions were suggested to be responsible for the different performance of these two spinel materials.

## Introduction

Recently, lithium-ion batteries (LIB) based on transition-metal oxide electrodes, typically in a spinel structure, have attracted extensive research interests for their remarkable electrochemical properties^1–9^. The capacity of these electrodes is typically 2–3 times larger than that of the graphite/carbon based electrode in the initial charge/discharge cycle. In order to understand the lithium storage mechanism in these electrodes, different characterization techniques have been utilized to provide a fundamental insight into the battery operation^10–13^. Benefiting from the ability to obtain real-time information on phase transition, metastale phase formation and change in microstructure, *in-situ* characterization is a powerful and favorable tool to study the behavior of electrodes in the battery cycle^14–16^. In the literature, the *in-situ* characterization for the electrodes has been carried out via transmission electron microcopy (TEM) and extended X-ray absorption fine structure **(**EXAFS) techniques, which often require tedious sample preparing processes or have to be conducted under special experimental conditions^15^. Moreover, most of the investigations were focused on the structural or morphological changes during the battery cycle, and less attention has been paid to magnetism evolution even though 3d transition-metal (TM) oxides often involve ferromagnetic properties. Although structural and morphological characterizations of the electrodes are necessary, magnetic measurement appears to be an accurate, sensitive, and convenient characterization method that provides information on the detailed atomic interaction during the intercalation/deintercalation of Li-ions. Additionally, it is one of the more adaptable measurement methods that can be applied to most materials under different temperature and external field conditions. Considering that the interaction between the electrode material and the Li-ions is definitely accompanied with change in electronic structure, *in-situ* magnetization characterization is a promising method to achieve a deeper understanding of battery operation. More importantly, this would also benefit research on the manipulation of magnetism via the guided motion of Li-ions.

The electrochemical control of physical properties like magnetism has been intensively investigated in materials with multiple functionalities^17–19^. For some spinel transition-metal oxides like ferrites, the coexistence of magnetism and ion storage ability make it possible to fabricate modulable devices controlled by Li-ions^20–23^. As electrodes for LIBs, TM oxides would experience a series of redox reactions during the battery cycle, which will change the state of the 3d electrons and their magnetic properties. Consequently, manipulation of magnetization by intercalation/deintercalation of Li-ions could be expected^24–27^. However, the structure transition from spinel to rock salt is irreversible, which means this modulation can only be operated before the phase transition. Another challenge is that the amount of Li-ions that can be inserted before the phase transition is closely related to the morphology, particle size, and other factors of the anode material^28^. These factors increase the complexity of the modulation process. Therefore, an in-depth and detailed understanding of the relationship between the magnetism change and lithiation process is currently lacking. Until now, there are few reports about the reversible control of ferromagnetism in spinel materials during lithium intercalation and deintercalation, and the explanation is mostly restricted to the change of chemical states^29–33^. This suggests that further research needs to be conducted to explore whether there are other modulating mechanisms during the battery cycle.

In this work, *in-situ* magnetic measurement was performed on spinel MnFe_2_O_4_ and γ-Fe_2_O_3_ electrode based LIBs. Reversible control of magnetization has been realized during the lithium intercalation/deintercalation. The electrochemical reaction process was investigated from the beginning of Li insertion into the spinel structure until the anode fully converted into other phases. Based on the results obtained from a variety of complementary analytical tools that were used to probe the structural, electronic, and chemical changes, a modulation mechanism focused on the magnetic ions’ migration in the lattice and induced magnetic coupling evolution during the battery operation is proposed.

## Results

### *Ex-situ* structural characterization

**Figure 1:**
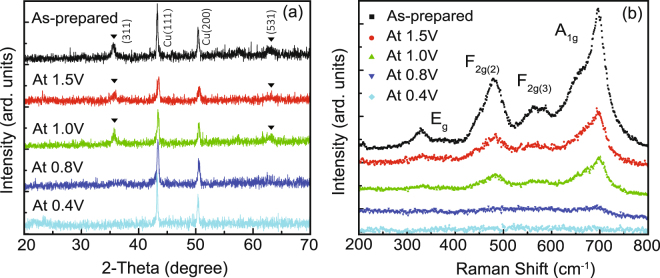
*Ex-situ* characterization of MnFe_2_O_4_ discharged to different voltage stage. (**a**) XRD patterns, (**b**) Raman spectra profiles.

*Ex-situ* structural characterization results including X-ray diffraction (XRD) patterns and Raman spectra profiles of MnFe_2_O_4_ are shown in Fig. 1. Note that the XRD results of the as-prepared electrode and the electrodes discharged to 1.5 V, 1.0 V, 0.8 V, and 0.4 V are given in Fig. 1a respectively. The two peaks at 43.3° and 50.4° can be attributed to Cu (111) and (200) peaks from the copper foil current collector. MnFe_2_O_4_ (311) and (531) peaks are marked by black triangles. As the diffraction patterns illustrate, the electrode maintains spinel structure until it is discharged to 1.0 V. When the cell is discharged below 0.8 V, the crystal structure collapses with the disappearance of the characteristic peaks and cannot recover even if the cell is charged back to 3.0 V (not shown here). This structural evolution is consistent with previous reports that the spinel structure changes into rock salt nanocrystals at low voltage and cannot be detected by XRD^34^. Fig. 1b shows a series of Raman spectra during the discharge process, in which all the peaks can be assigned to normal spinel structure. Further investigation indicates that the peaks of the prepared sample are asymmetric (or dissociated) with a shoulder on the low energy side. Each peak can be decomposed into a doublet, which is a typical characteristic of the inverse spinel structure^35^. At a microscopic level, this implies that the Fe^3+^ ions are distributed both in A- and B-sites instead of only B-sites as in a completely normal spinel structure. With lithium intercalation, the symmetry of the A_1g_ mode—which comes from symmetric stretching of oxygen atoms along Mn-O (and Fe-O) bonds in the tetrahedral coordination—improves a little, implying a redistribution of the metal ions in oxygen interstices. All of the vibration modes decrease slightly from 1.5 V to 1.0 V, and when the cell is discharged to 0.8 V the vibration modes vanish entirely, which is consistent with the XRD results.

### *Ex-situ* magnetic and electrochemical characterizations

**Figure 2:**
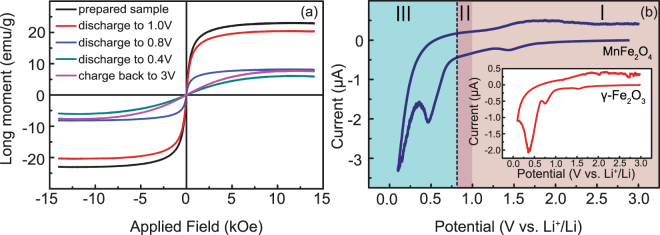
*Ex-situ* magnetic and electrochemical characterizations. (**a**) Magnetic hysteresis measurement results of MnFe_2_O_4_ at different voltage stage. (**b**) The CV curves of MnFe_2_O_4_ electrode between 0.01 and 3.0 V at a scan rate of 0.1 mV/s. The inset gives the outcome of γ-Fe_2_O_3_. Three regions are divided according to the structure variation in the discharge process. (I) remaining spinel structure, (II) changing into rock-salt structure, (III) reduced into metals.

*Ex-situ* magnetic and electrochemical characterization results including hysteresis loops and cyclic voltammetry (CV) curves are shown in Fig. 2. The magnetic measurement results of MnFe_2_O_4_ are shown in Fig. 2a. Compared with the as-prepared MnFe_2_O_4_, the saturation magnetization demonstrates a slight decrease when the cell is discharged to 1.0 V. When the electrode material is further discharged to 0.8 V, the saturation magnetization shows a dramatic drop. Considering the structural change above, the decrease could be attributed to the structural damage of spinel. The saturation shows further decrease when the electrode is discharged to 0.4 V, even though no notable structural change was detected by either XRD or Raman spectra. The saturation field increases noticeably at 0.4 V, implying a possible formation of nano-sized Fe particles^31^. Another possibility is that this phenomenon is caused by some nano-sized spinel residual which could show spin glass**–**like behaviors and a high saturation field. When the electrode is recharged to 3.0 V, the saturation increases slightly, but cannot be restored to the prepared state. This implies that the electrode has changed into a mixture of iron and manganese oxides after the delithiation process instead of the original MnFe_2_O_4_ phase. On the other hand, the slight saturation rise may indicate that some Fe ions have been oxidized to magnetic Fe_3_O_4_ rather than Fe_2_O_3_ upon lithium extraction, which has also been proved by the *in-situ* TEM observation^15^.

Figure 2b shows the CV curve of the MnFe_2_O_4_ electrode with the Li metal as a counter/reference electrode at a voltage scan rate of 0.1 mV/s between 0.01 and 3.0 V. A discharge plateau from 3.0 V to 1.7 V can be observed. The first distinguished reduction peak occurance around 1.5 V, which is occasionally observed in spinel electrodes, is usually attributed to the formation of a solid electrolyte interphase (SEI) layer^36^ and the decomposition of the electrolyte^15^. However, this feature is mainly observed within the stability window of the presently used solvent. It is more likely due to the reduction of surface functional groups, and could also be ascribed to an electrochemical grinding of the particles as a result of internal strains or a second phase formation caused by side reactions^28^. Further experimental evidence is needed to clarify this reduction peak. The broad cathodic and anodic peaks at 0.5 and 1.9 V respectively could be explained by the reduction and oxidation between the corresponding oxides and the metallic elements. Since the first discharge plateaus of Fe_2_O_3_ and MnO are usually located at around 0.8 and 0.2 V, the reduction peaks could be overlapped to form a broad peak around 0.5 V^14,15^. γ-Fe_2_O_3_ demonstrates similar electrochemical behavior except that there is an additional reduction peak around 0.75 V, which is indicative of structural destruction. An ideal maghemite contains only trivalent iron ions and has many unoccupied interstitial sites in the spinel structure, which may make the spinel to rock salt structural phase transformation much easier and the lithium intercalation peak more pronounced.

According to the structure change, the Li insertion process in spinel can be divided into three steps. For the case of MnFe_2_O_4_, the discharging stages have been highlighted in different colors in the CV profile in Fig. 2b. In region I, the material remains in the spinel phase. In region II, the insertion of Li-ions leads to the formation of the defective/distorted NaCl-type structure. In region III, the insertion leads to a reduction of Fe^2+^ and Mn^2+^ to metals and the formation of Li_2_O. Moreover, according to the Raman spectra in Fig. 1b, MnFe_2_O_4_ is not a single-phase composite, which implies that slight occupancy of Fe^3+^ at the oxygen tetrahedron sites is inevitable. Two different processes may happen at the same time in region I^14^. First, Li atoms reduce Fe^3+^ on the tetrahedral 8a sites to Fe^2+^ and force them to move to the adjacent octahedral 16c sites. Second, more Li atoms force the Mn^2+^ ions in the A sites to move to the 16c sites while an appropriate amount of Fe^3+^ ions on the 16d sites are reduced. However, typical characterization techniques such as XRD cannot give direct proof for the suggested reaction mechanism due to the following combined factors: low resolution for a complicated electrode system, incomplete conversion reactions, and electrochemically induced pulverization and amorphization. To overcome these limitations, an *in-situ* technique is used in this study to understand the exact conversion mechanism, especially in region I.

### *In-situ* magnetic measurements

**Figure 3:**
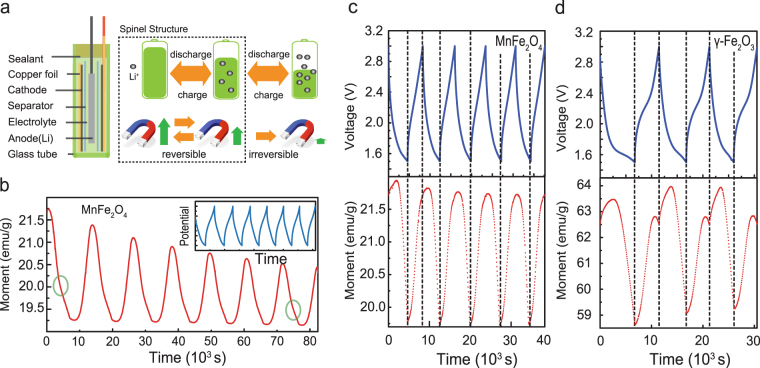
*In-situ* magnetic results performed simultaneously with the electrochemical discharge/charge processes. (**a**) Illustrations of the lithium-battery for *in-situ* magnetic measurement and the schematic of magnetism variation during the discharge/charge process. (**b**) The magnetism variation of MnFe_2_O_4_ in the range from 3.0 to 1.0 V. The modulation value of saturation magnetization decay obviously. The inflection point of variation trend is marked by green circle. The inset gives the charge/discharge curves. (**c**), (**d**) The magnetism variation of MnFe_2_O_4_ and γ-Fe_2_O_3_ in the range from 3 to 1.5 V.

The schematic structure of the miniature lithium battery for *in-situ* magnetic measurement in a Superconducting Quantum Interference Device (SQUID) is shown in Fig. 3a. For the device fabrication, a glass tube was chosen as electrolyte compartment since it exhibits low magnetic susceptibility and high homogeneity. A chemically resistant epoxy resin was used to seal the device, with two feedthroughs connecting the Li anode and the cathode. The battery was loaded into the SQUID for *in-situ* magnetic measurement, while a cell test instrument was connected for the battery cycle. *In-situ* magnetic measurement was conducted within the voltage range in which the material remains in its spinel structure, and the saturation can be changed reversibly.

**Figure 4:**
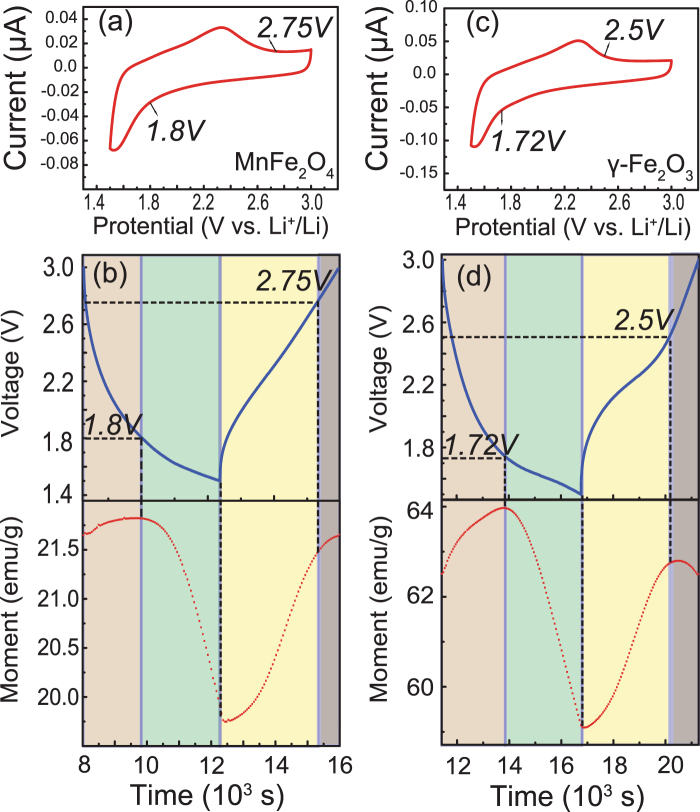
(**a**), (**c**) The CV curves of MnFe_2_O_4_ and γ-Fe_2_O_3_ in the range from 3 to 1.5 V. (**b**), (**d**) The enlarged image of the second charge/discharge cycle for MnFe_2_O_4_ and γ-Fe_2_O_3_. The processes are divided by different magnetic variation trend, and marked in different color.

The measurement was carried out with a 1 T magnetic field applied to the sample and the magnetic moment was recorded every 50 seconds under a current density of 50 mA/g with a galvanostatic discharge and charge cycle. As shown in Fig. 3b, when the battery is cycled in the voltage window from 1.0 to 3.0 V, a magnetic moment fade can be observed with insignificant capacity shrinking. This fading is caused by some irreversible electrochemical/chemical reaction in the battery operation, which may relate to the first reduction peak in the CV profile as mentioned above. From the inset, we can find the voltage decreases smoothly under such a discharge current, and it is hard to find the discharge plateau corresponding to the reduction peak. However, the magnetism measurement is more sensitive and we can detect an inflection point on every dropping line of the magnetization measurement, which always happens around 1.5 V. When the voltage window was set between 1.5 and 3.0 V, a significant improvement of the magnetism stability was observed as shown in Fig. 4c,d.

A detailed investigation of the stable magnetization variations is shown in Fig. 4c,d. For the case of MnFe_2_O_4_, the saturation first rises and then drops in the discharge process, and for γ-Fe_2_O_3_ the increase is more apparent. In the few reports about magnetism modulation of γ-Fe_2_O_3_ and Fe_3_O_4_ in Li batteries, the Neel linear model was utilized to explain the magnetic variation, attributing the change to valence variation or electrochemical reaction^29,30^. However, simply considering the valance change from Fe^3+^ (5 μ_B_) to Fe^2+^ (4 μ_B_), the maximum saturation variation per formula is only 20%, which is smaller than some experimental results. If the TM ions’ migration from tetrahedron to octahedron is taken into account, the magnetism enhancement caused by the ions’ reposition would be much larger than the decline caused by the ions’ reduction^29^. Then the magnetization after Li intercalation would become larger than the as-prepared state, which conflicts with the experimental results. Therefore, there must be some other factor that dominates the variation.

In order to find additional details of the magnetism saturation variation in the voltage window between 1.5 and 3.0 V, we performed the CV tests at 0.1 mV/s for both MnFe_2_O_4_ and γ-Fe_2_O_3_ to make a comparison with the enlarged ferromagnetism modulation profiles as shown in Fig. 4. It clearly shows that the magnetism varies slowly on the charge/discharge plateaus and changes quickly around the cathodic and anodic peaks. With the electrodes remaining their spinel structure, the peaks in the discharge and charge processes, which implies a quick insertion of Li-ions, represent a variation of ionic and electronic conductivity.

## Discussion

Nonmagnetic ion doping effect in spinel ferrite has been reported by Gorter, and his work provides a helpful reference to deduce the mechanism of magnetism variation in our experiments^37^. In a Neel linear model, the spinel magnetic ions are antiferromagnetic coupled by a super-exchange effect. The coupling (AB interaction) between the ions in oxygen tetrahedron (A-site) and the ions in oxygen octahedron (B-site), is much stronger than the coupling strength between the ions in A-sites (AA interaction) or B-sites (BB interaction). The ions between tetrahedron and octahedron sites are coupled in an antiferromagnetic way, forcing the ions within the tetrahedron or octahedron sites to align in a ferromagnetic way. The total magnetism is caused by the unbalanced magnetic moment between the tetrahedron and octahedron sites. As mentioned above, the Li intercalation would make the TM ions in A-sites transfer to the adjacent B-sites. If the moment of A-sites drops, the magnetism per formula should rise. However, if A-sites are occupied mainly by nonmagnetic ions, the AB super-exchange coupling will decrease and the BB antiferromagnetic interaction will play the dominate role. Then the magnetism per formula would decrease.

**Figure 5:**
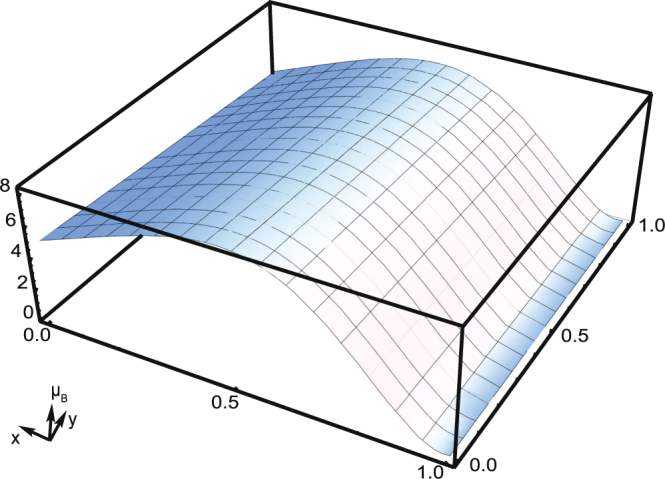
Calculation results based on a statistical model. The magnetic moment of MnFe_2_O_4_ per formula has been given as a function of Li ions intercalation. X and y stands for the average amount of intercalated Li ions per chemical formula respectively in the tetrahedral and octahedral sites.

Gilleo has made a super-exchange calculation in spinels which contain randomly incomplete linkages based on the statistic model^38^. A similar calculation has also been made to help understand the magnetism change in the Li-ions’ insertion process (see details in Supporting Information). The magnetic moment of MnFe_2_O_4_ as the function of Li-ions intercalation per formula is shown in Fig. 5. This profile exhibits first a rising then a falling trend, which resembles our experimental results. The investigation also found that the magnetization is very sensitive to the Li-ions’ intercalation in the tetrahedral sites. Consequently, it was believed that the linkage breaking of the AB interaction should play a more important role than the ions’ reduction in the magnetism variation.

**Figure 6:**
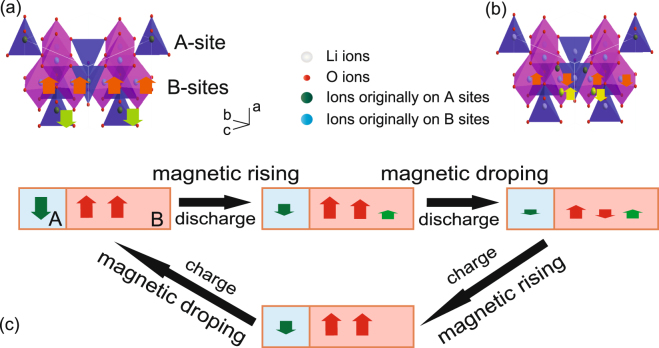
The schematic of the spinel magnetic coupling variation in the Li insertion/extraction process between 3.0 V and 1.5 V. (**a**) State of the high magnetization material. Magnetic ions in oxygen tetrahedrons (A) and octahedrons (B) are coupled antiferromagnetically between each other, and arranged ferromagnetically in their own sites. (**b**) State of the low magnetization material. The ions in A sites have been transferred to B sites, and the magnetic ions are coupled antiferromagnetically. (**c**) The magnetism variation mechanism of reversible process in the discharge/charge cycle.

According to the discussions above, a possible mechanism of the magnetic evolution in the reversible control process is given in Fig. 6. When Li-ions are inserted into the electrode material, they first reduce the trivalent ions in the A-sites, which means Fe^3+^ for MnFe_2_O_4_ and γ-Fe_2_O_3_, and then force them to migrate to the nearby empty B-sites. According to the Neel linear model, a tetrahedral magnetism decrease would lead to an increase of the compound total magnetization. Additionally, the increased magnetic ions in the B-sites would also cause an enhancement of the entire magnetic moment. As a result, a rising saturation occurs at the beginning of the discharge process. Considering there are more Fe^3+^ ions participating in the reduction of γ-Fe_2_O_3_ tetrahedron sites, it is reasonable to deduce that its magnetism increase is more intense than that in MnFe_2_O_4_. The ions migration also explains the Raman symmetric improvement in MnFe_2_O_4_. However, this magnetic ion loss in the tetrahedral sites would weaken the super-exchange coupling between the A and B-sites, making the BB interaction more pronounced. As a result, coupling within the octahedrons starts to transform to an antiferromagnetic order. At the beginning of the discharge stage, because the TM ions in the A-sites can still maintain the original magnetic order, the enhancement effect exceeds the fading one. After being discharged beyond the critical point, the remaining ions can hardly maintain the original magnetic arrangement, making the BB super-exchange effect gradually dominate the coupling. In addition to the ions’ migration, as the insertion process goes on, Li-ions cause the reduction of ions in octahedron sites, which aggravates the magnetic fading further. As a result, the magnetism decreases sharply at the end of the discharge process.

In the charge process, lithium ions extracting first makes the bivalent ions migrate from B-sites to A-sites, which means that the antiferromagnetic coupling between A and B-sites is gradually strengthened. Considering that Mn^2+^ is difficult to oxidize to higher valence, the oxidizing should mainly happen in B-sites from Fe^2+^ to Fe^3+^, and this magnetic moment variation would result in a magnetization increase. As the magnetic ions in A-sites increase, the super exchange effect between AB sites becomes stronger than the effect between BB sites, and ions in A-sites or B-sites become ferromagnetically aligned again. On the second stage of charging, the magnetism increase becomes slow for MnFe_2_O_4_ and even decreases in case of γ-Fe_2_O_3_. It is most likely caused by the A-sites ions’ oxidation from bivalent to trivalent, making the magnetization of the whole compound decline. Considering that more Fe ions occupy the tetrahedral sites in γ-Fe_2_O_3_ than that in MnFe_2_O_4_, this decrease should be clearer in γ-Fe_2_O_3_ than in MnFe_2_O_4_.

## Conclusions

We have fabricated miniature lithium-batteries using MnFe_2_O_4_ and γ-Fe_2_O_3_ as electrodes. Both batteries show reversible electrical control of magnetization over a value of 10% in the voltage range between 1.5 V and 3.0 V. The electrical and magnetic behaviors of spinel electrodes in the Li inserting/extracting process, especially the initial reversible range, were investigated. An electrode property modulation mechanism based on magnetic ions migration and exchange coupling evolution has been proposed to explain the saturation magnetization change. This finding contributes the in-depth understanding of Li-ion induced physical property changes in spinel-structured materials and potentially lead to the advancement of modulation-based device fabrications.

## Methods

Commercial MnFe_2_O_4_ and γ-Fe_2_O_3_ nanoparticles with average size of 20 nm (Aladdin Industrial Co.) were used to prepare the electrodes. 80 wt% active materials, 10 wt% acetylene black and 10 wt% polyvinylidene fluoride (PVDF) binder were mixed and dissolved in N−methyl pyrrolidinone (NMP). The slurry was coated on a copper foil current collector. Then it was dried in a chamber electric furnace at 70 °C for 10 hours to form a working electrode. The miniature Li battery cells were assembled into a small glass tube with lithium metal as the counter electrode. A Celgard2325 microporous polypropylene membrane was used as separator, and LBC3015B (Shenyang Kejing Auto-instrument Co.) as electrolytes. The assembling process was carried out inside an argon-filled glove box.

The crystal phase of the samples was characterized by X-ray diffraction (XRD). Charge/discharge experiments were conducted using a CT2001A cell test instrument (LAND Electronic Co.). An electrochemical workstation (RST5202) was used to study the cyclic voltammetry (CV) performance. The *Ex-situ* magnetic measurement was carried out at room temperature using an alternating grating gradient magnetometer (AGM). Raman spectra were obtained by a confocal Micro-Raman spectrometer with an excitation wavelength of 515 nm (Olympus FV500, Japan). The as-prepared working electrode and the electrodes collected at different discharge voltage after washed in dimethyl carbonate (DMC) were measured respectively. The *in-situ* magnetic measurements were performed simultaneously with the discharging/charging processes in a Quantum Design superconducting quantum interference device (SQUID) magnetometer.

## Electronic supplementary material

Supplementary information
