# Supplementary material for: Reversible control of the magnetization of spinel ferrites based electrodes by lithium-ion migration
Source: Sci Rep. 2017 Oct 2;7:12554. doi: 10.1038/s41598-017-12948-6 (PMC5624968; doi:10.1038/s41598-017-12948-6)
Supplement: Supplementary file 1 — Supplementary information [file 41598_2017_12948_MOESM1_ESM.pdf]

SUPPLEMENTAL MATERIAL

Reversible control of the magnetization of spinel ferrites based electrodes by lithium-ion migration

Guodong Wei, Lin Wei, Dong Wang, Yanxue Chen, Yufeng Tian, Shishen Yan, Liangmo Mei and Jun Jiao

In this supplemental material, additional details on the super-exchange calculation based on a statistical model are provided. It is known that ions in octahedral sites and tetrahedral sites are bridged with each other through an oxygen ion, which is to say that each oxygen ion is coordinated with one of the octahedral ions and one of the tetrahedral ions. However, when magnetic ions are replaced by nonmagnetic Li-ions, a number of linkages will be incomplete. Magnetic ions, which do not interact with other magnetic ions, are excluded from participation in ferrimagnetism. In addition, with a large amount of iron ions replaced by nonmagnetic ions, the possibility that a magnetic ion may interact with only one other magnetic ion must be taken into account. An ion with a single linkage cannot be active in a cooperative interaction with the remainder of the ions, as is required in ferrimagnetism, because it cannot relay the orientation information contained in the single linkage to any other magnetic ions. The interaction chain through a magnetic ion must be uninterrupted in order to actively participate in ferrimagnetism. Consequently, a single linkage should be discarded and the ion thus linked also excluded from participation in ferrimagnetism.

For an ion of coordination n, the probability that it only links with m magnetic ions is:

$P_n(m) = \frac{n!}{m!(n-m)!} k^{n-m}(1-k)^m$, where k is the occupying probability of nonmagnetic ions.

Therefore, the probability E that an ion be linked with none, or at most one, of the magnetic ions is

$$E(k) = \sum_{m=0}^{1} P_n(m) = \mathrm{n}k^{n-1} - (n-1)k^n.$$

A magnetic ion actively participates in ferrimagnetism only if it interacts with two or more magnetic ions in a different coordination, so the probability is $1 - E(k)$.

For a compound of spinel structure, E_{ka} and E_{kb} of the octahedral and tetrahedral sublattices are:

$$E_{\mathrm{ka}} = 6{k_a}^5 - 5{k_a}^6,$$

$$E_{\mathrm{kb}} = 12{k_b}^{11} - 11{k_b}^{12}.$$

As mentioned above, the Li intercalation would make the TM ions in A-sites transfer to the adjacent B-sites. It is convenient to use the following formula to represent a spinel structured lithiation $MnFe_2O_4$:

$$(\mathrm{Li^+}_x\mathrm{Mn^{3+}}_{1-x})[\mathrm{Mn^{2+}}_x\mathrm{Fe^{2+}}_{x+y}\mathrm{Fe^{3+}}_{2-x-y}\mathrm{Li^+}_y]O_4.$$

In a unit cell, the ions of the formula which are in octahedral sites are enclosed by brackets while those in tetrahedral sites are enclosed by parentheses. To simplify the formula, it can be written:

$$(\mathrm{Li}_x M(A)_{1-x})[M(B)_{2+x}\mathrm{Li^+}_y]O_4,$$

where the A- and B-site moment per magnetic ion is:

$$\mu_a = 5\mu_B,\ \mu_b = \frac{4(x+y)+5(2-y)}{\mathrm{x}+(x+y)+(2-\mathrm{x}-y)} = \frac{10+4x-y}{2+x}\mu_B.$$

So, the moment per formula unit is:

$$B = \mu_b(2+x)[1-E_{\mathrm{ka}}] - \mu_a(1-x)[1-E_{\mathrm{kb}}]$$

$$= (10-y+4x)(1-6x^5+5x^6) - 5(1-x)[1-12\left(\frac{y}{2+x+y}\right)^{11} + 11\left(\frac{y}{2+x+y}\right)^{12}]\mu_B.$$
